# Special Issue “Emerging Viruses: Surveillance, Prevention, Evolution, and Control”

**DOI:** 10.3390/v12030306

**Published:** 2020-03-11

**Authors:** Jônatas Santos Abrahão, Luciana Barros de Arruda

**Affiliations:** 1 Laboratório de Vírus, Instituto de Ciências Biológicas, Departamento de Microbiologia, Universidade Federal de Minas Gerais, Belo Horizonte 31270-901, MG, Brazil; 2 Departamento de Virologia, Instituto de Microbiologia Paulo de Góes, Universidade Federal do Rio de Janeiro, Rio de Janeiro 21941-902, RJ, Brazil

Emerging viruses represent a major concern for public health offices. Climate changes, the international migration of people and products, deforestation, and other anthropogenic activities (and their consequences) have been historically and continuously related to the emerging and re-emerging of new viruses, triggering an increasing number of notified outbreaks, epidemics, and pandemics. In this very popular Special Issue (Emerging Viruses: Surveillance, Prevention, Evolution, and Control), we are proud to have received a total of 70 manuscript submissions, with an acceptance rate 54.29% (38 papers) from colleagues from different parts of the world, working on a wide range of topics.

The deadline for this Special Issue concurred with the emergence of a new human pathogen, the coronavirus SARS-CoV-2, which is spreading in dozens of countries around the world, reinforcing the impact of emerging viruses in public health and the global economy. Interestingly, a considerable number of articles discussing diversity, transmission, and pathogenesis of human and animal infection by respiratory viruses, including coronaviruses, draws the attention of those who follow this issue. Coronaviruses, in particular, are addressed in three papers. Kandeil et al. [1] demonstrate a high seroprevalence of MERS coronavirus in adult dromedary camels from Asia and Africa, and Alsaadi et al. [2] characterize a fusion peptide in the spike protein of MERS-CoV. Avian coronaviruses are also addressed in the article from Fan et al. [3], which reveals the changes in genetic diversity, dominant genotypes, and selection pressure on the bronchitis virus (IBV) circulating in yellow chickens in China.

A few papers access genetic and pathogenic features of avian influenza viruses, which have caused important outbreaks of different bird species and serious losses to the poultry industry, besides being associated to sporadic human infections. Yeo et al. [4] present the characterization of a novel avian influenza A (H2N9) from a wild duck in Korea. Song et al. [5] isolate H5N6 isolates from waterfowls in China, which appear to be multi-reassortant among different genotypes of avian influenza viruses. They demonstrate relevant host–virus features in chickens and mice, including systemic replication and contact transmission between chickens, and upregulation of PRR in different organs in chicken and mice. Mei et al. [6] also reveal that goose-origin H5N6 avian influenza viruses have different pathogenicity and transmissibility in chickens. Yu et al. [7] present a comparative analysis between highly and low pathogenic H7N9 AIV strains. They show that both virus isolates were shed through respiratory and digestive routes in inoculated chickens and could be transmitted to naïve animals; however, the emerging H7N9 highly-pathogenic avian influenza presents a stronger pathogenicity and transmissibility. Sun et al. [8] present data on the H9N2 avian influenza viruses currently circulating in South China and Song et al. [9] compare biological and genetic features of three H9N2 avian influenza in chicken and mice models, showing that different isolates may have distinct replication and transmissibility efficiencies. 

H1N1 swine influenza features are also addressed in this issue. Song et al. [10] compare phylogenetic and pathogenic features of two swine influenza H1N1 viruses isolated from Shandong. By phylodynamics analyses, Adam et al. [11] suggest an increased selection of influenza H1N1/pdm09 clade 6b residues and other high mortality mutants in India.

Arboviruses represent a hot-topic as well. Maia et al. [12] present the discovery of novel viruses in mosquitoes from Brazilian wetlands, including viruses from nine different families: *Iflaviridae*, *Phenuiviridae*, *Rhabdoviridae*, *Flaviviridae*, *Reoviridae*, *Chuviridae*, *Circoviridae*, *Partitiviridae*, and *Totiviridae*. The natural coinfection of *Aedes aegypti* by chikungunya and dengue type 2 virus in Brazil is also reported by Aragão et al. [13] The influence of the environment on chikungunya virus transmission in Senegal is revealed by Sow et al. [14]. Barbosa Costa et al. [15] describe a possible silent circulation of the Saint Louis encephalitis virus among humans and equids in Brazil. Rezende et al. [16] present an interesting case of late-relapsing hepatitis after acute yellow fever virus infection. Arbovirus diagnosis, in particular Zika virus diagnosis, is also discussed. Pérez-Olmeda [17] evaluates the performance characteristics of the LIAISON XL Zika Capture IgM II. Versiane et al. [18] describe the identification of B-cell epitopes with potential use to discriminate dengue from zika infections in serological assays.

We received several papers on veterinary viruses. Yu et al. [19] present data on the prevalence and co-infection of fowl adenovirus. Meng et al. [20] identify and characterize a novel fowl adenovirus and perform an immunoprotective evaluation of an inactivated FAdV-4 vaccine. The emergence of avian orthoavulavirus 13 in wild migratory waterfowl in China and related genetic features are presented by Fei et al. [21]. Lu et al. [22] report a novel subtype of bovine hepacivirus in China. Águeda-Pinto et al. [23] present a genetic characterization of a recombinant myxoma virus in the *Lepus granatensis*. Data on the immunogenicity in rabbits of virus-like particles from a rabbit haemorrhagic disease virus is presented by Miao et al. [24]. Malossi et al. [25] sequence and analyze the genome of different equine infectious anemia viruses, revealing that Brazilian strains share a monophyletic cluster. Sozzi et al. [26] describe the isolation and genome sequencing of a pestivirus from aborted lamb fetuses in Italy. Chen et al. [27] evaluate the genetic diversity of porcine circovirus 3 in China. Morandini et al. [28] report a novel adélie penguin circovirus from Antarctica. Chen et al. [29] present insights on the phylogeny and virulence of porcine reproductive and respiratory syndrome virus variants. 

Other relevant research papers are also published. Garry and Garry [30] present in depth proteomics computational analyses on the antennavirus glycoprotein complex. Ribeiro et al. [31] present data on a nymphalid-infecting group I alphabaculovirus. Spitz et al. [32] present relevant data on the first complete genome sequence of hepatitis C virus subtype 2b from Latin America. Silva et al. [33] demonstrate that a given TREX1 polymorphism is associated with higher proviral load, lower inflammatory cytokine levels, and is more frequent in HAM/TSP symptomatic patients infected with HTLV-1. Moutelíková et al. [34] show the emergence of rare bovine–human reassortant DS-1-Like rotavirus A strains in human patients in the Czech Republic. Duarte et al. [35] present the virome of different wild birds and mammals from Brazilian cerrado, showing sequences of members of *Adenoviridae*, *Anelloviridae*, *Circoviridae*, *Caliciviridae*, and *Parvoviridae* families, complete or nearly complete genomes of known anelloviruses, circoviruses, and parvoviruses, as well as putative novel species.

At last, we also received reviews on emerging viruses. Martins et al. [36] discuss the existence of antivirals against the chikungunya virus in nature. Wubshet et al. [37] review data on the foot and mouth disease virus in Ethiopia from 2008 to 2018. Judson et al. [38] examine nosocomial transmission of emerging viruses via aerosol-generating medical procedures.

Although this Special Issue was prepared by *Viruses* in collaboration with the Brazilian Society for Virology, we are very grateful for having received submissions from several countries and hundreds of colleagues. We believe that the success of this Special Issue is the consequence of the great job done by *Viruses* staff and all authors that decided to submit their research here. On behalf of the Brazilian Society of Virology, thank you!
